# Patient-specific *in silico* 3D coronary model in cardiac catheterisation laboratories

**DOI:** 10.3389/fcvm.2024.1398290

**Published:** 2024-07-05

**Authors:** Mojtaba Lashgari, Robin P. Choudhury, Abhirup Banerjee

**Affiliations:** ^1^ Institute of Biomedical Engineering, Department of Engineering Science, University of Oxford, Oxford, United Kingdom; ^2^ Division of Cardiovascular Medicine, Radcliffe Department of Medicine, University of Oxford, Oxford, United Kingdom

**Keywords:** x-ray coronary angiography, coronary artery disease, catheterisation laboratory, patient-specific model, *in silico* medicine, segmentation, 3D reconstruction, blood flow simulation

## Abstract

Coronary artery disease is caused by the buildup of atherosclerotic plaque in the coronary arteries, affecting the blood supply to the heart, one of the leading causes of death around the world. X-ray coronary angiography is the most common procedure for diagnosing coronary artery disease, which uses contrast material and x-rays to observe vascular lesions. With this type of procedure, blood flow in coronary arteries is viewed in real-time, making it possible to detect stenoses precisely and control percutaneous coronary interventions and stent insertions. Angiograms of coronary arteries are used to plan the necessary revascularisation procedures based on the calculation of occlusions and the affected segments. However, their interpretation in cardiac catheterisation laboratories presently relies on sequentially evaluating multiple 2D image projections, which limits measuring lesion severity, identifying the true shape of vessels, and analysing quantitative data. *In silico* modelling, which involves computational simulations of patient-specific data, can revolutionise interventional cardiology by providing valuable insights and optimising treatment methods. This paper explores the challenges and future directions associated with applying patient-specific *in silico* models in catheterisation laboratories. We discuss the implications of the lack of patient-specific *in silico* models and how their absence hinders the ability to accurately predict and assess the behaviour of individual patients during interventional procedures. Then, we introduce the different components of a typical patient-specific *in silico* model and explore the potential future directions to bridge this gap and promote the development and utilisation of patient-specific *in silico* models in the catheterisation laboratories.

## 1 Introduction

Coronary artery disease (CAD) is a pathological process characterised by atherosclerotic plaque accumulation in the epicardial coronary arteries. There are several clinical manifestations of this disease, including chronic stable angina and acute coronary syndromes. According to the World Health Organisation’s global health estimates and global burden of disease data (estimates for 2019), CAD is the most commonly diagnosed heart disease worldwide. It is estimated around 200 million people are living with CAD, and it kills an estimated 9 million people each year (1).

Investigation of CAD includes functional evaluations, such as stress echocardiography, perfusion stress magnetic resonance imaging, and nuclear scintigraphy in myocardial perfusion imaging. In addition, computed tomography coronary angiography and invasive x-ray angiography can be used to evaluate the coronary arteries directly. Invasive x-ray coronary angiography is particularly valuable in patients with more severe disease, informing treatment decisions including the possibility of revascularisation through percutaneous coronary intervention (PCI) or bypass surgery. Coronary angiography provides high-resolution images of the coronary arteries that are widely used for stent implantation. It is often augmented with additional techniques, such as pressure wire evaluation of fractional flow reserve (FFR), intravascular ultrasound (IVUS), and intravascular optical coherence tomography (IOCT).

Although x-ray angiography is one of the most invaluable tools, it does have some drawbacks. First of all, it is an invasive procedure with potential vascular injury, haemorrhage, and embolisation. Furthermore, this procedure involves the use of x-ray contrast media that can cause or exacerbate renal dysfunction and cause adverse allergic reactions. For example, the US National Cardiovascular Data Registry reported that 7.1% of patients undergoing elective and urgent coronary intervention experienced contrast-induced acute kidney injury (2). In addition, there are issues of ionising radiation exposure for both the patient and the operator (3). Finally, the interpretation of the angiographic images is partially subjective and is prone to misinterpretation or variable interpretation (4). It is estimated that 70% of treatment decisions still depend on the visual assessment of angiographic stenosis within clinical settings, which has limited accuracy (about 60%–65%) in predicting FFR < 0.80, as reported by Hae et al. (5).

To overcome such vagaries, additional physiological studies including FFR or intravascular imaging are often utilised. For example, Jones et al. (6)’s large observational study confirms that IVUS and IOCT-guided PCI reduces in-hospital major adverse cardiac event rates and improves long-term survival when compared with standard x-ray angiography-guided PCI. However, they are expensive and time-consuming (7).

Patient-specific *in silico* models have shown their capability to enhance qualitative assessment by introducing quantitative elements into the diagnostic, interventional, and prognostic processes in different cardiovascular diseases (8–10). With *in silico* techniques, coronary arteriography could be more accurately assessed in real-time, with fewer views, less radiation, less contrast, and easier administration, all of which would benefit clinical practice. Using artificial intelligence (AI)-assisted *in silico* models, cardiologists only need two series of x-ray angiography sequence to generate the 3D structure of a coronary arterial tree, as shown by (11), thus reducing the time of x-ray exposure and dye injection while providing an accurate quantitative assessment. Additionally, it offers the computation of haemodynamic metrics such as FFR non-invasively, through blood flow simulation over the 3D structure, using mechanistic (12, 13) or data-driven (14) approaches.

**Figure 1:**
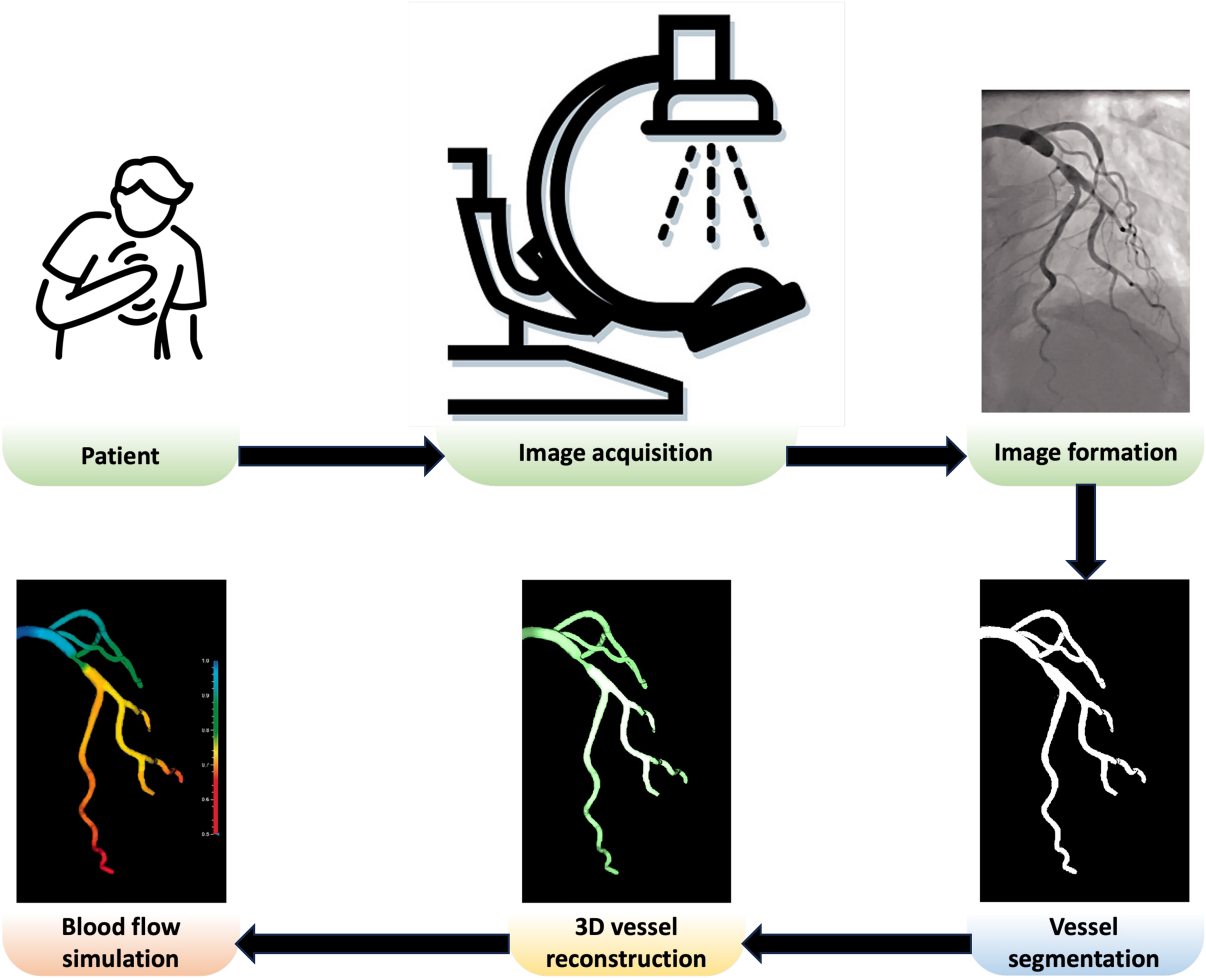
Overview of a patient-specific *in silico* model in the cath. lab. Adapted with permission from (15), © The Foundation Acta Radiologica 2021, https://doi.org/10.1177/0284185120983977.

In this paper, we review the key components needed to create a patient-specific *in silico* coronary model, as shown in Figure 1. After acquiring comprehensive and high-quality x-ray angiography sequences of a patient, the coronary arteries can be segmented using automated approaches discussed in Section 2. Detailed anatomical 3D digital twins of the patient’s coronary tree can then be generated using the techniques discussed in Section 3. In the next steps, the digital twins of coronary arteries can be used for blood flow simulations, detailed in Section 4, which can be applied for computions of quantitative haemodynamic metrics to detect coronary stenoses and assess their severity (Section 5). Finally, Section 6 comprehensively discusses how patient-specific *in silico* models can be utilised to optimise the patientcare pathway in the catheterisation laboratory (cath. lab.). The paper concludes in Section 7.

## 2 Coronary vessels segmentation

3D reconstruction of the coronary arteries, discussed in detail in Section 3, often relies on the back-projection model-based methods, which require accurate skeletal representation and radius of coronary arteries as the inputs. The skeleton and radius of the vessels are typically obtained by segmenting the blood vessels. This section provides a comprehensive overview of different coronary vessel segmentation methods used for this purpose.

### 2.1 Non-temporal methods

#### 2.1.1 Traditional statistical and machine learning based

• **Image thresholding** is a simple image segmentation method in which grayscale images are turned into binary images by categorising each pixel according to its intensity level concerning a threshold value. To improve the result of image thresholding in x-ray angiography, the coronary vessels are usually enhanced by utilising different imaging filters (16–19).
• **Vessel tracking** is another form of segmentation that involves extracting a path along a vessel from a designated starting point (20). Some techniques focus on isolating individual paths with defined start and endpoints, while others can identify the entire vessel tree and adeptly manage vessel branching (21–25).
• **Edge detection** identifies and extracts a set of points representing changes in brightness on an image, commonly referred to as an edge contour, arising from variations in grayscale between vessels and the background (26–29).
• In **region growing** method, seed pixels are used to create regions, and neighbouring pixels meeting specific criteria are added to those regions (30). To improve the results of region growing for vessel segmentation, different approaches have been proposed such as incorporating directional information (31–33), integrating with different methods such as random forest (34), and variable searching method of the pixels (35).
• **Graph-cut** uses a graph model to represent the image, where nodes represent pixels and edges represent the relationships between pixels in a graph. It divides the image into segments based on certain criteria, such as colour or intensity, to find the optimal cut in the graph (36). Hernandez-Vela et al. (37), Sun et al. (38), Mabrouk et al. (39) developed automated multi-scale vessel extraction algorithms using the graph-cut method.
• **Fuzzy inference** uses the human-like reasoning style and offers potent and adaptable universal approximations, allowing interpretable IF-THEN rules (40). Sun et al. (41) used fuzzy mathematical morphology operations to extract coronary arteries, while Shoujun et al. (42) proposed a tracking approach that relied on both probabilistic vessel tracking and fuzzy structure pattern inference.
• In **deformable models**, a segmentation objective function (or energy function) is optimised through the calculus of variation. Image data constructs an energy function; minimising it yields segmentation results. These models use the original image for initial and boundary value problems. The contour, initially set as the desired region’s boundary, evolves based on geometric image regions (43). Different variations of deformable models have been used for coronary vessels segmentation, such as parametric active contours (44, 45), geometric active contours (32, 46, 47), gradient vector flow active contour (48), region-based active contour (49), etc.
• Methods based on **machine learning** models leverage intricate algorithms and training on diverse datasets to enhance the ability to discern intricate coronary vessels structures from a complex background of x-ray angiography. The machine learning methods used for coronary vessels segmentation include marginal space learning paradigm and probabilistic boosting trees (50), random forest (51), robust principal component analysis (PCA) (52, 53), etc.

#### 2.1.2 Neural network based

Neural networks, modelled after the human brain, consist of interconnected neurons organised into layers. During training, they adjust connection strengths between neurons to minimise prediction errors using a method called backpropagation. Once trained, neural networks make predictions by passing new data through the network based on learned patterns. Success depends on training data quality, network architecture, and parameter selection. Neural networks generally segment images by classifying pixels into specific categories, such as objects or boundaries. This process involves leveraging patterns and features within the image data to accurately delineate different regions. One of the oldest applications of neural networks for identifying coronary vessels in x-ray angiograms was done by Sun (54), pioneering the use of neural networks in medical imaging for precise vessel localisation and analysis.

**Figure 2:**
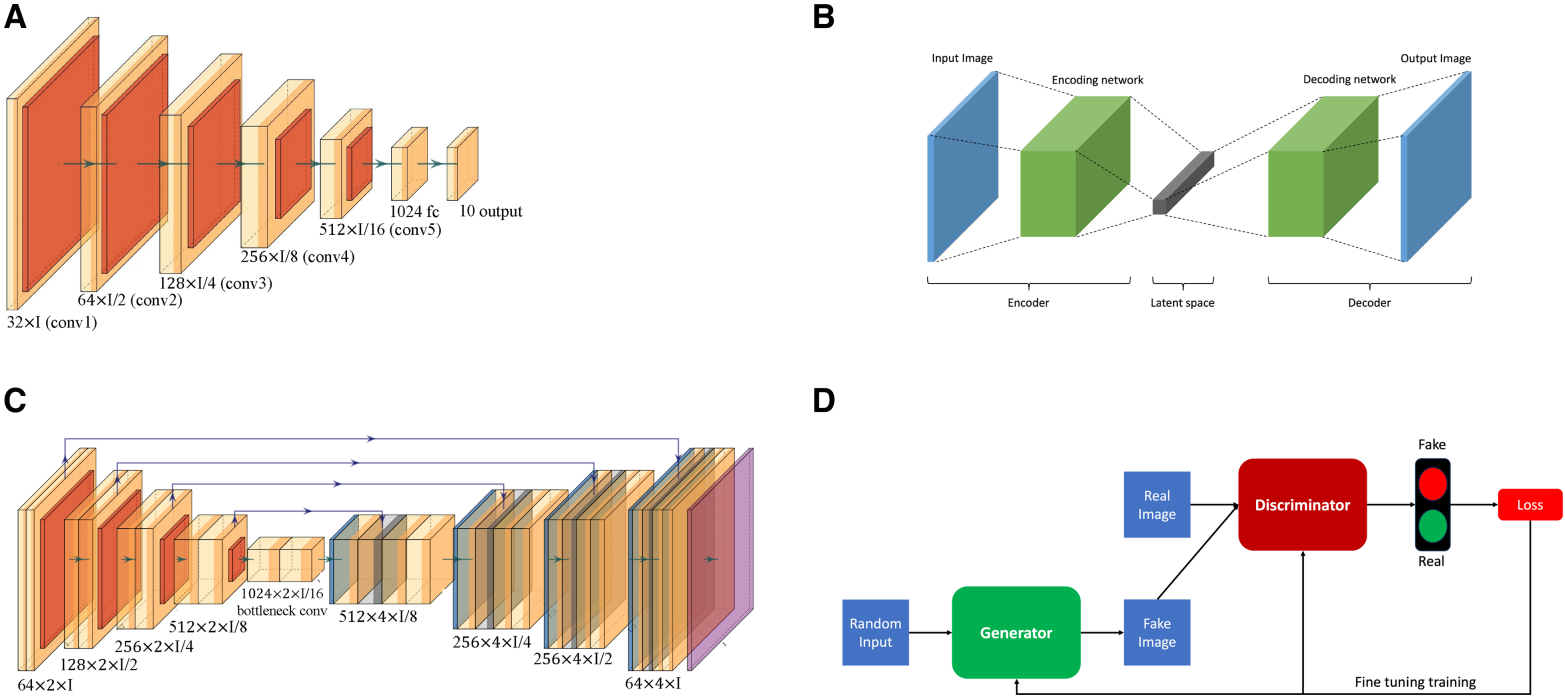
Overview of the basic neural network architectures used for coronary vessels segmentation: (**A**) Convolutional neural network (CNN); (**B**) Encoder-decoder; (**C**) U-Net; and (**D**) Generative adversarial network (GAN).

• **Convolutional neural networks** (CNNs), a type of deep learning model, have been employed for tasks of coronary vessels segmentation in multiple studies (55–58). CNNs, as shown in Figure 2A, utilise layers to learn hierarchical features through convolutional operations, pooling, and fully connected layers, enabling automatic and adaptive spatial feature learning from the input images.
• **Encoder-decoder**, illustrated in Figure 2B, is another type of deep learning neural network used for coronary vessel segmentation (59, 60). Encoding involves passing an image through a series of convolutional and pooling layers, e.g., Figure 2A. In these layers, spatial dimensions are downsampled while capturing the important features, thus extracting hierarchical features while condensing the input image. In the decoder, the spatial dimensions are gradually reconstructed using upsampling operations based on the feature map from the encoder.
• **U-Net** architecture, as shown in Figure 2C, is an example of encoder-decoder architecture designed to segment images. It was introduced by Ronneberger et al. (61) and has since become a popular and effective neural network used for coronary vessels segmentation (62–65). It is named after its distinct U-shaped structure and differs from other encoder-decoder networks because it uses skip connections to connect the corresponding layers of encoding and decoding, thus preserving fine-grained details during segmentation.
• **Adversarial learning** is another type of neural network applied for coronary vessels segmentation (66). This network involves training a model against adversarial examples generated to deceive the model. The model learns to be more robust by experiencing and adapting to these adversarial inputs. A popular branch of adversarial learning is generative adversarial networks (GANs), which have been used for vessel segmentation in x-ray angiography (67, 68). A GAN consists of two structures, a generator and a discriminator, as presented in Figure 2D. The generator generates data instances, while a discriminator evaluates them. Both are trained simultaneously, with the generator aiming to produce realistic data and the discriminator aiming to distinguish between the real and the generated data.
• **Attention mechanism** enables models to make predictions while focusing on specific details of coronary vessels in images (69). This mechanism is usually incorporated into various deep neural networks such as encoder-decoder (70), U-net (71), and adversarial network (66).
• **Ensemble deep learning** is intended to enhance the model’s generalisation, robustness, and accuracy by leveraging the diversity of multiple models. To improve the overall performance of vessel segmentation, ensemble deep learning models combine vessel predictions from multiple individual neural network models such as combining style transfer with dense extreme inception network and convolution block attention (72), EfficientNet with U-Net (73), gradient-boosting decision trees with deep forest classifiers (74), ensemble encoder-decoder networks (75), U-Net with DenseNet-121 (76), and bi-directional ConvLSTM algorithm with U-Net and DenseNet models (77).

More recently, advanced deep learning architectures namely Nested U-Nets or UNet++ (78), graph neural network (79), etc. have been utilised for acheiving state-of-the-art performance for coronary vessels segmentation in x-ray angiography.

### 2.2 Temporal methods

All the methods discussed in the previous subsection used a single frame to segment the coronary vessels. Some studies in the literature also utilised multiple invasive coronary angiography (ICA) frames to capture temporal information for vessels segmentation. These studies aimed at mitigating noise and motion, enhancing overall contrast, and were robust to variations in image quality, illumination, and other artifacts. Temporal methods have been applied to improve segmentation quality over a variety of techniques, including vessel tracking (80), region growing (81), graph cut (82, 83), machine learning (84), as well as neural network approaches of CNN (85, 86), encoder-decoder (87), U-Net (88, 89), ensemble learning (90), etc.

## 3 3D reconstruction of coronary arteries from ICA images

A patient-specific model relies heavily on 3D geometry of the coronary vessels or the whole coronary arterial (CA) tree. This helps cardiologists and medical professionals better understand the anatomy, structure, and any potential abnormalities in the arteries during the intervention, and guide catheters and devices to the target area with greater accuracy. Additionally, the reconstructed 3D CA tree model allows for personalised treatment plans tailored to the specific anatomy of the patient through simulating different treatment scenarios, leading to optimised outcomes and reducing the risk of complications. However, creating an accurate 3D model of coronary arteries is a sensitive task and crucial to a successful intervention as well as a personalised treatment plan. For example, Solanki et al. (91) using arterial phantom models showed that minor reconstruction errors led to clinically significant inaccuracies in “virtual” fractional flow reserve (vFFR) computation.

Çimen et al. (92) reviewed the leading methods for reconstructing the 3D surface of coronary arteries using high-contrast x-ray angiography. In this section, we explain briefly the categories introduced by Çimen et al. (92) along with a brief review of the most recent 3D CA tree reconstruction approaches.

### 3.1 Back-projection based methods

Though several different approaches have been proposed for coronary artery reconstruction in the literature, back-projection based methods remain the most common. Back-projection methods fall into the model-based reconstruction categories, which aim to create a 3D/4D binary model of coronary arteries, typically comprising a centerline and sometimes the vessel surface. In back-projection modelling, the CA tree is constructed by projecting two-dimensional (2D) information derived from ECG-gated projection images. There are two types of methods: methods that rely on 2D feature matching and methods that use back-projection of vesselness responses (92).

**Figure 3:**
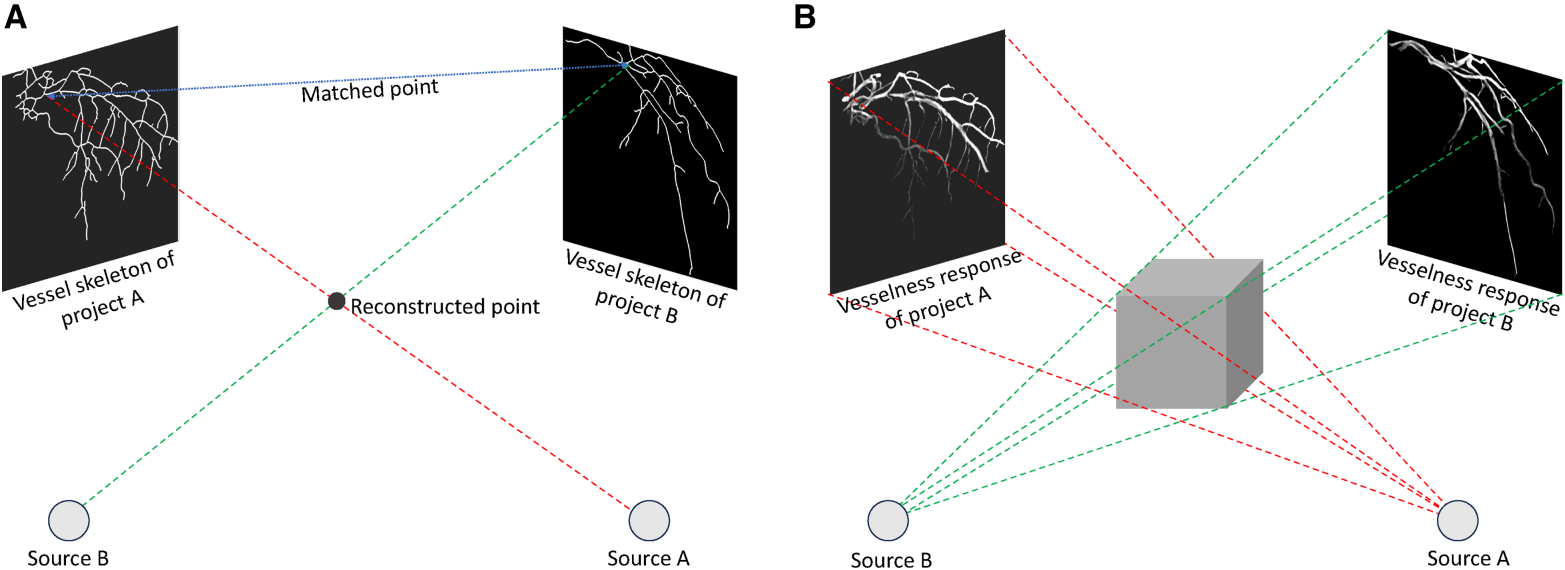
Back-projection methods to reconstruct 3D coronary arteries: (**A**) Techniques relying on 2D feature matching establish correspondences between centrelines in distinct 2D views and perform reconstruction through triangulation, (**B**) Approaches centered on the back-projection of vesselness response generate a 3D volumetric vesselness response from 2D vesselness responses for subsequent processing.

**Methods based on 2D feature matching**, depicted in Figure 3A, begin by segmenting artery centerlines and identifying key structures such as bifurcations within projection images. Using epipolar geometry, correspondences between centerlines are established between different views, and computer vision algorithms are used to reconstruct 3D points representing the CA tree. The accuracy of these methods relies heavily on segmentation accuracy during centerlines extraction. Recently, Çimen et al. (92) represented 3D coronary artery centerlines as a mixture of Student’s *t*-distributions and performed a maximum-likelihood estimation of model parameters using 2D x-ray image segmentation. Unberath et al. (93) enhanced reconstruction quality by effectively removing erroneously reconstructed points on the centerline. Vukicevic et al. (94) used a robust genetic algorithm optimiser to identify calibration parameters for x-ray angiography views. A partial-matching approach was applied to establish correspondences between frames in x-ray acquisitions, and the same matching method was applied to reconstruct vessel centerlines efficiently. Galassi et al. (95) reconstructed the 3D centerlines by intersecting surfaces from matching branches from 2D views. Then, the 3D luminal contours were created by interpolating computed 3D boundary points with non-uniform rational basis splines. In another work, Banerjee et al. (11) first reduced angiographic motion artifacts for rigid and non-rigid motion (96, 97), and then used an innovative point-cloud based approach to 3D vessel centerline reconstruction by iteratively minimising reconstruction error. These methods are beneficial to non-calibrated systems since they can easily incorporate the estimation of geometry parameters that relate to the projection images used for reconstruction. The vascular start/end and bifurcation points, which are extracted during segmentation, are often used for this purpose. Although some works tried to match these corresponding points (98–103), most of the 3D reconstruction methods need clinicians to manually find these corresponding points in the projected images.

In contrast, in **methods based on back-projection of vesselness responses**, shown in Figure 3B, 2D projection images are used to calculate vesselness responses, such as binary segmentation (104), tubularity response (105), and distance map to centreline (106). These responses are then back-projected based on imaging geometry to generate 3D volumetric vesselness responses. Following this, coronary artery reconstruction is conducted using segmentation methods. One of the drawbacks of these methods is that they may require more rotational x-ray angiogrms in order to generate an accurate 3D reconstruction.

### 3.2 Forward-projection based methods

Another type of model-based reconstruction methods is forward-projection that employs 3D models that adapt to vessel structures in 2D x-ray projections. The forward-projection reconstruction often relies on 3D parametric active contour methods, where external and internal energy, computed from images, are used to adjust 3D active contours (107–109). In CA tree reconstruction, every artery branch has its active contour model, which presents a challenge in designing energy components. Cong et al. (107) compared common deformable model based methods, namely potential energy (110), gradient vector flow (111), and generalised gradient vector flow (112), on a series of experiments on phantom and clinical data.

### 3.3 Tomographic reconstruction

Tomographic reconstruction creates coronary artery volumes directly from x-ray coronary angiography images. As opposed to binary model based reconstruction, it provides information on x-ray absorption coefficients, as well. Due to the minimal knowledge they require about the CA trees, these methods accommodate atypical anatomies (e.g., collaterals, tortuous branches). As a result, they provide more detailed vessel surface information without any preprocessing or manual inputs (113–115).

The drawbacks of these methods are they assume pre-acquisition calibration of the x-ray imaging system and typically require more x-ray images with wider angular coverage than modelling based reconstructions. For coronary artery branches to be visible, these methods require precise isocentering and consistent injection of contrast. Moreover, they ignore the propagation of contrast agents over time, assuming constant contrast distribution over time. Finally, these approaches typically require more computational resources than model based reconstructions. Also, it is often necessary to hold breath during coronary angiography to minimise respiratory motion to reconstruct tomographic images (92).

### 3.4 3D+time (4D) model based reconstruction

Model based methods can be extended to 4D coronary artery reconstructions. The basic 4D strategy involves independent 3D reconstructions for each cardiac phase, which requires vessels segmentation for each phase. To avoid this, temporal constraints which penalise differences between adjacent phases have been utilised, and temporal correspondences have been established through branch or tree-matching algorithms (116–118).

## 4 Simulation

The final component for completing the patient-specific model in cath. lab. is to simulate hemodynamics in coronary arteries. The patient-specific model of blood flow in the coronary arteries using computed tomography has been well established due to fewer challenges in the creation of a 3D model of coronary arteries (12, 119). Some of the technological developments for blood flow simulation using computed tomography can be used directly over 3D vascular models from x-ray angiography, as these methods are often independent of imaging techniques. The following subsections describe the developments for coronary blood flow simulation.

### 4.1 *In silico* flow computation approaches

**Figure 4:**
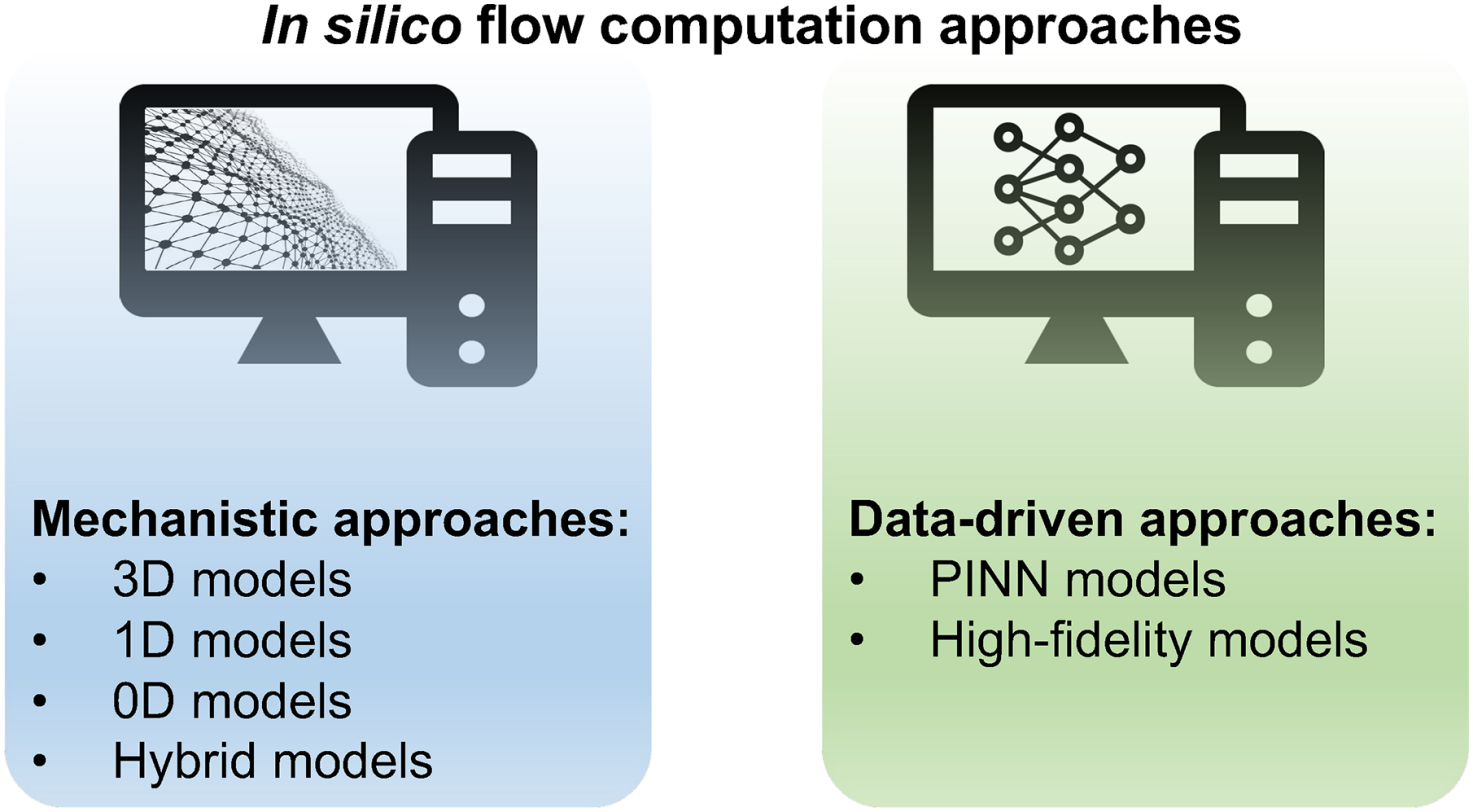
Overview of *in silico* flow computation approaches.

The Navier-Stokes equations are a set of partial differential equations that represent the physics of a fluid dynamic. However, they are complex partial differential equations, which are difficult to solve analytically and computationally expensive to simulate accurately. There are two distinct approaches of mechanistic modelling and data-driven modelling (Figure 4) to simulate blood flow based on the Navier-Stokes equations, each with its own set of characteristics and advantages.

#### 4.1.1 Mechanistic approaches

This approach involves formulating mathematical equations that represent these processes. This approach is commonly used in fields where a deep understanding of the system is available, and where the goal is to gain insights into the underlying processes, optimise system performance, or test hypotheses. The mechanistic models to solve Navier–Stokes equations can be divided into four groups (12, 13).

(a) **3D models** use numerical methods such as finite elements to solve Navier–Stokes equations. Using this approach, circulation geometry can be accurately represented, 3D pulsatile flow (including turbulence) can be captured, and complex blood and vessel material models can be incorporated (120).
(b) **1D models** are created by averaging the Navier-Stokes equations over a vessel’s cross-section. These models ignore non-axial velocity components, assume an axial velocity profile across locations of vessels, and maintain constant pressure across the vessel cross-section. However, these models are invalid near side branches, bifurcations, or diseased segments, especially for serial lesions or lesions at branches and bifurcations (120–123).
(c) The **0D model** or lumped parameter circulation model, developed by Sagawa et al. (124), consolidates spatially varying properties into discrete components. Considering the flow steady, axisymmetric, unidirectional, and vessel segments as circular cylinders, this model simplifies fluid resistance in vessels to a single resistive element. It often results in a high level of inaccuracy in blood flow in diseased coronary arteries, where no steady or unidirectional flow occurs or no axisymmetry or circular shape to vessel segments exist (122, 124–126).
(d) The **hybrid models** aim to reduce computational time while providing more accurate simulation results. It includes a combination of the different mechanistic approaches such as 0D and 1D models (123, 125) and 1D and 3D models (13, 127–129).

#### 4.1.2 Data-driven approaches

Data-driven approaches extract patterns, relationships, or trends from observed data without explicitly considering underlying physical or mechanistic principles. The technique is often used for modelling complex, unknown, time-consuming, or difficult-to-model physical processes. There is, however, a lack of sufficient training data for data-driven methods (130). To address the lack of experimental data, two solutions exist: enhancing deep learning through physics-based losses, known as physics-informed neural networks (PINN) (131–134), or conducting high-fidelity *in silico* simulations to implicitly make the model sensitive to the underlying physics (135–139).

In the application of coronary blood flow using a large high-fidelity dataset, Itu et al. (140) introduced a machine learning model to predict fractional flow reserve (FFR), trained on a large database of synthetically generated coronary anatomies with flow parameters like velocity computed using the mechanistic approaches. Carson et al. (141) compared the performance of three AI models – feed-forward neural network (FFNN), long short-term memory, and multivariate polynomial regression, to measure FFR. Based on a 1D physics-based model, algorithms were trained and compared on a single vessel, multi-vessel network, as well as a virtual patient database, demonstrating the outperformance of a FFNN over two other methods in all cases. Gao et al. (142) proposed TreeVes-Net, a recurrent neural network (RNN) that captures geometric details for blood-related representation using a tree-structured representation encoder. This tree-structured RNN creates long-distance spatial dependencies, enhancing coronary flow modelling. Xie et al. (143) suggested a physics-informed graph neural network for FFR assessment, incorporating morphology and boundary conditions as inputs to learn conditioned features. In another work, Zhang et al. (144) proposed a PINN including a morphology feature encoder and an attention network to simulate the pressure and velocity along the centerline of the vessels based on the morphology features of coronary arteries.

### 4.2 *In silico* flow computation based on x-ray angiography images

**Mechanistic model** developed by Morris et al. (145), termed virtual FFR (vFFR), was one of the first to accurately predict coronary artery disease based on patients’ FFR using only x-ray angiography images. The initial vFFR model necessitated over 24 hours of computation, employing a fully transient, 3D-0D coupled model. Faster methods were introduced in 2017 (146) and 2023 (147), yielding results in just 3 min and less than 30 s, respectively, while maintaining the accuracy of a full 3D model (148). Recently, more studies have been developed to provide angiography-derived FFR based on mechanistic modelling (149–156).

With **Data-driven models**, Zhao et al. (157), Xie et al. (143) proposed deep neural networks based on CNN and graph neural network to compute the FFR and coronary flow reserve (CFR), respectively.

## 5 Quantitative hemodynamic metrics for coronary artery assessment

Functional metrics for coronary lesion severity assessment can be established through the development of patient-specific *in silico* models. Incorporating computational simulations with clinical data allows to gain a more detailed understanding of the complex dynamics inside coronary arteries, enabling better assessment metrics. According to several studies (158–161), quantitative measurements of arterial stenosis severity reduce unnecessary surgeries and cardiac events in patients with coronary artery disease. This section discusses the most important metrics used for this purpose.

**Figure 5:**
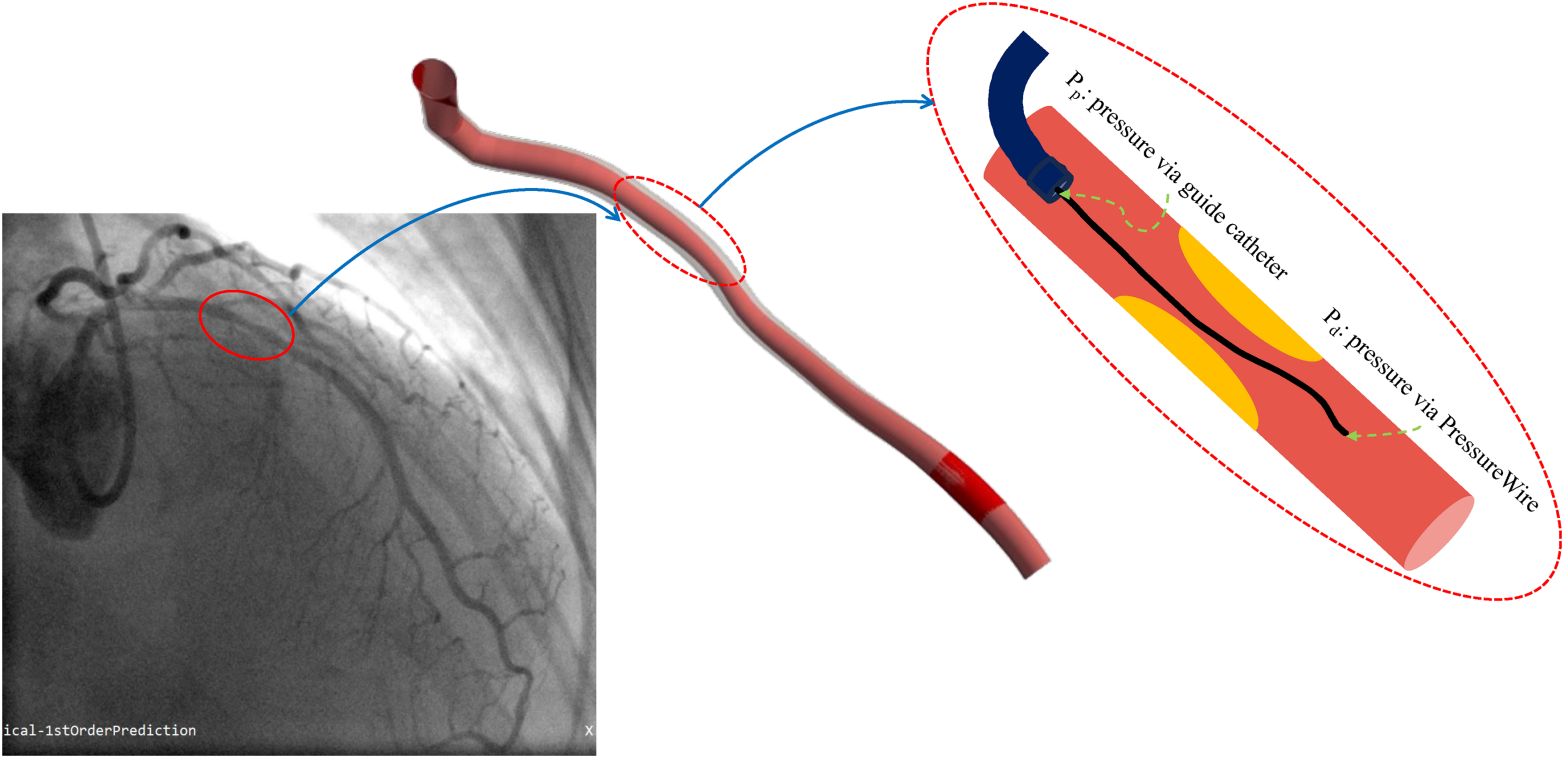
Measuring blood pressure before and after stenosis.

(i) **Fractional flow reserve (FFR)** traditionally has been used for assessing the hemodynamics of coronary arteries. As part of the cardiac catheterisation procedure, a special wire is threaded through the coronary arteries, equipped with a pressure sensor. FFR is then calculated by comparing blood pressure before and after the stenosis as shown in Figure 5. Using this ratio, clinicians can determine how much blood flow to the heart has been impeded by the narrowing. FFR can provide valuable insights into whether coronary stenosis requires intervention, such as angioplasty or stent placement, or can be treated medically (162).
(ii) **Coronary flow reserve (CFR)** is another diagnostic metric in the decision-making process for coronary interventions. It is calculated by comparing coronary blood flow during maximal vasodilation and at rest. Three factors influence it: vascular resistance in the small and large coronary arteries, myocardial resistance, and factors that affect blood composition. CFR provides valuable information for evaluating coronary artery dilation ability and determining whether coronary interventions are necessary (163). By comparing the CFR of a stenotic coronary artery to a reference segment, such as a non-stenotic segment or one with minimal disease, relative CFR (rCFR) can be calculated. It is particularly useful when evaluating the functional implications of a coronary artery by comparing the blood flow through a particular stenosis to that of another less affected artery (158, 164, 165).
(iii) **Index of microcirculatory resistance (IMR)** was introduced in 2003, where a pressure wire uses its sensor as a thermistor and measures temperature. The tool functions as a thermometer and measures the mean transit time of room-temperature saline injected into a coronary artery using a thermodilution curve. As part of the test, pressure and temperature are measured in the heart’s small vessels, both at rest and at maximum blood flow. It allows the clinician to determine how well blood flows in the heart’s small vessels (166–168).
(iv) **Instantaneous wave-free ratio (iFR)** is a test using a special catheter to check the pressure in the heart’s blood vessels. It looks at pressure during both the wave-free period and the entire cardiac cycle. This helps doctors see how a blockage affects blood flow. One of the advantages of iFR is that it doesn’t need adenosine, a medicine used in other tests to stress the heart and check blood flow (169–171).
(v) **Resting full-cycle ratio (RFR)** is a new index that is used to evaluate the significance of coronary vessel lesions. In the cardiac cycle, it is defined as the lowest ratio of distal pressure to aortic pressure, measured at rest without the introduction of hyperemia (172–174).

Table 1 summarises the quantitative hemodynamic metrics for coronary artery assessment, along with the mathematical formula.

**Table 1:** Summary of quantitative hemodynamic metrics for coronary artery assessment.

Formula	Usage
$FFR=\frac{P_{d}}{P_{p}}$	To determine functional significance of a coronary stenosis (162).
$CFR=\frac{F_{h}}{F_{r}}$	To determine whether coronary arteries can dilate and accommodate increased blood flow (163).
$rCFR=\frac{CFR_{\mathrm{Stenosis}}}{CFR_{\mathrm{Reference}}}$	To evaluate the impact of a specific stenosis on blood flow compared with a healthier or less affected reference region (158).
$IMR=\frac{P_{d}}{T_{\mathrm{mn}}}$	To provide information about the status of microcirculatory resistance.
$iFR=\frac{P_{d_{wave-free}}}{P_{a_{wave-free}}}$	Similar to FFR, without the need to administer a hyperemic agent, such as adenosine (169).
$RFR=\frac{P_{d}}{P_{a}}$	Similar to FFR, without the need to administer a hyperemic agent, such as adenosine (173).

$P_{d}$, pressure measured at the distal of a stenosis; $P_{p}$, pressure measured at the proximal of a stenosis; $F_{h}$, coronary blood flow during maximal hyperemia; $F_{r}$, coronary blood flow at rest; $T_{\mathrm{mn}}$, mean transit time of a specific flow and temperature; $P_{d_{\text{wave-free}}}$, $P_{d}$ measured at rest during a wave-free period; $P_{a_{\text{wave-free}}}$, $P_{a}$ measured at rest during a wave-free period; $P_{a}$, aortic pressure.

## 6 Discussion

The development of patient-specific models in the cath. lab. can enhance patient care and improve outcomes in various ways. A patient-specific model can provide detailed insight into the anatomy and pathology of a patient’s vascular system. Clinicians can use this information to plan and customise cardiac interventions like angioplasty, stent placement, or other procedures. Furthermore, physicians can anticipate challenges by simulating procedures on patient-specific models before they are performed. In addition, patient-specific models help with the selection and size of medical devices, such as stents and catheters, based on accurate vessel dimensions and measurements. Additionally, patient-specific models enhance research by exploring new treatment strategies, testing innovative devices, and understanding underlying physiology.

As illustrated in Figure 1, a variety of medical image analysis methods should be employed to develop patient-specific models, including segmenting coronary arteries, reconstructing 3D geometry, and simulating blood flow to compute physiological biomarkers to detect and assess stenosis severity. There are potential sources of uncertainty in each stage of the modelling process, which can potentially propagate to the subsequent stages, resulting in an unreliable simulation result. For instance, Solanki et al. (91) demonstrated that the errors arising from the epipolar line projection method used to reconstruct 3D coronary anatomy from x-ray angiography images are small but result in clinically relevant errors in vFFR simulation, amounting to approximately 40% of the total error associated with vFFR. In the following paragraphs, we discuss the potential sources of uncertainty at each stage of the analysis.

**Segmentation:** According to Table 2, deep learning methods have significantly improved coronary vessels segmentation accuracy. However, there exist three potential challenges that limit the generalisation performance and result in discontinuity in segmented vessels. The first challenge is the imaging artifacts such as weak contrast between coronary arteries and the background, unknown vessel tree shape, and shadows of overlapping body structures. Secondly, due to the acquisition of a complex 3D structure in 2D projection planes, the coronary vessels overlap on x-ray angiography images, making segmentation and vessel delineation difficult, especially in regions where vessels are close together. Last but not least, the x-ray angiography captures contrast agents flowing through vessels during dynamic imaging. Hence, segmentation methods often face challenges with temporal changes and vessel appearance variations throughout the cardiac cycle. The recent advances in AI and deep learning have shown promising results in addressing some of these shortcomings, though they are often limited by the training datasets.

**Table 2:** Summary of the best performance of different coronary vessels segmentation methods.

Study	Method	Dataset size	Dice	Sensitivity	Specificity	Precision	Accuracy
Felfelian et al. (17)	Thresholding	50 (Test)	72.79	74.92	98.32	–	97.09
Tsai et al. (24)	Tracking	20 (Test)	–	96.70	96.30	–	96.30
Mabrouk et al. (39)	Graph-cut	91 (Test)	75.60	76.60	–	77.60	–
Lv et al. (46)	Deformable model	4 (Test)	76.24	72.33	–	80.59	–
Jin et al. (52)	PCA	223 (Test)	76.97	71.25	83.95	–	–
Zhu et al. (58)	CNN	73 (Train), 36 (Test)	88.40	87.30	–	90.10	–
Iyer et al. (59)	Encoder-decoder	370 (Train), 92 (Test)	86.40	91.80	98.70	–	98.30
Yang et al. (64)	U-Net	2,642 (Train), 660 (Test)	89.60	89.30	–	90.60	–
Hamdi et al. (67)	GAN	100 (Train), 50 (Test)	81.18	81.09	98.11	81.26	96.55
Tao et al. (70)	Attention mechanism	104 (Train), 30 (Test)	–	87.70	97.89	–	97.29
Gao et al. (74)	Ensemble method	104 (Train), 26 (Test)	87.40	90.20	99.20	85.70	–

**3D reconstruction:** In recent years, 3D reconstruction methods for coronary arteries based on x-ray angiography have gone through significant advances, though they still face some challenges. First, the 3D surface reconstruction can be irregular due to non-orthogonal contours to the vessel centerline and difficulties defining its cross-sectional shape. The information available from 2D x-ray angiography projections is often limited, as they can only provide limited information at a finite number of projection planes and may not provide a complete representation of the vessel geometry. Secondly, movements in coronary arteries mainly due to cardiac and respiratory motions create difficulties in establishing correspondences between 2D segmented vessels and, as a result, affect the vessel centerlines reconstruction. Since correspondence is commonly based on epipolar constraints, significant vessels overlap and foreshortening may impact their performance.

The 3D vascular geometry can also be reconstructed using IVUS and IOCT, which are both intravascular imaging technologies to capture cross-sections of coronary arteries. In contrast to x-ray angiography, IVUS and IOCT images reveal external elastic membrane and plaque materials in addition to the coronary lumen. These approaches, however, are used to image a single branch of non-bifurcated vessels, and they considerably increase patient-care costs (175, 176). X-ray angiography, on the other hand, provides a very accurate image of the entire coronary arterial tree with minimal invasion, which makes it safer and more useful for automated 3D reconstruction of coronary vessels.

**Blood flow simulation:** The development of sophisticated computational fluid dynamics techniques has allowed researchers to model blood flow in complex coronary arteries with greater accuracy. There still exist some challenges and limitations. Limitations in segmenting small or terminal vessels limit coronary blood flow simulation in larger epicardial vessels. The boundary conditions at the outlet of the terminal vessel approximate downstream arterial circulation behaviour, and the lack of segmenting terminal vessels leads to a wrong boundary conditions assignment which invalidates assessment of the disease’s impact on myocardial blood flow (177, 178). Gamage et al. (179) showed that side branches downstream of stenosis result in a lower FFR, while those upstream have minimal impact. Moreover, to estimate FFR accurately, side branches with a diameter greater than one-third of the main vessel diameter should be taken into account. In addition, it is crucial to consider patient-specific boundary conditions and the associated uncertainties. If the associated parameters of the 3D inflow velocity profile cannot be specified, the domain flow field can be significantly impacted (180). Further research should try to calculate patient-specific boundary conditions by estimating the blood velocity in coronary arteries using cine x-ray angiographic sequence (181).

## 7 Conclusion

While coronary angiography remains a vital diagnostic tool in the assessment of coronary artery disease, the limitations inherent in the current interpretation methods call for a paradigm shift. Patient-specific *in silico* models, with their ability to simulate and analyse individualised data, present a promising avenue for advancing interventional cardiology. By addressing the challenges highlighted in this paper and accordingly embracing these models in catheterisation laboratories, we can unlock the full potential of *in silico* modelling. The integration of patient-specific *in silico* models into routine practice has the potential to revolutionise treatment optimisation, providing clinicians with valuable insights and enhancing the precision of interventions. Future research and development efforts should focus on bridging the existing gap and promoting the widespread adoption of these models in the cath. lab. settings.
